# Emerging Deep-Sea Smart Composites: Advent, Performance, and Future Trends

**DOI:** 10.3390/ma15186469

**Published:** 2022-09-17

**Authors:** Haiyi Zhou, Pengcheng Jiao, Yingtien Lin

**Affiliations:** 1 Institute of Port, Coastal and Offshore Engineering, Ocean College, Zhejiang University, Zhoushan 316021, China; 2 Engineering Research Center of Oceanic Sensing Technology and Equipment of Ministry of Education, Zhejiang University, Zhoushan 316021, China

**Keywords:** deep-sea composite materials, deep-sea exploration technology, smart composites, self-diagnosis, self-healing, self-powered

## Abstract

To solve the global shortage of land and offshore resources, the development of deep-sea resources has become a popular topic in recent decades. Deep-sea composites are widely used materials in abyssal resources extraction, and corresponding marine exploration vehicles and monitoring devices for deep-sea engineering. This article firstly reviews the existing research results and limitations of marine composites and equipment or devices used for resource extraction. By combining the research progress of smart composites, deep-sea smart composite materials with the three characteristics of self-diagnosis, self-healing, and self-powered are proposed and relevant studies are summarized. Finally, the review summarizes research challenges for the materials, and looks forward to the development of new composites and their practical application in conjunction with the progress of composites disciplines and AI techniques.

## 1. Introduction

With the economic development and population growth, the world faces extreme resources shortage. Since land and offshore resources are gradually becoming deficient, deep-sea energy exploitation has become a hot trend in recent decades [1]. Ocean energy, also named “blue energy”, has the advantages of pollution-free, wide distribution, and convenient collection. Blue energy harvesting devices based on friction generation technology rekindle the popularity of the research on the most concerned marine renewable energy [2]. Deep-sea resources are important strategic resources for the future sustainable energy, including petroleum, natural gas, and minerals. In order to better use these resources, the core issue that needs to be addressed is the stable operation of the resource extraction equipment in harsh marine environments, such as low temperature and high pressure due to the water depth [3]. At present, countries all over the world attach great importance to the technology research related to deep sea energy exploitation, for example, deep-sea mining devices, oil drilling platforms, submersible equipment, etc., as well as the special materials used in these deep-sea engineering [4]. In case of carrying out the exploration of marine resources, underwater acquisition and delivery systems for mining units and drilling platforms, as well as marine submersibles, present many new challenges to the operating conditions and functional requirements of exploration equipment [5,6]. For example, the superposition of extremely high seawater pressure and the structural stress of equipment itself, resulting in the horrible working conditions of the equipment. As another example, the deficient oxygen in abyssal conditions has a significant effect on the surface passivation of the material, accelerating the corrosion, or increasing the cracking tendency [7]. The adaptability of materials to the deep-water environment is an important foundation to ensure the stable operation of marine exploration equipment. Therefore, theoretical and practical research on deep-sea materials occupies an important position in the field of deep-sea resource exploration [8]. Smart materials are the fourth generation of materials after natural materials, synthetic polymer materials, and artificially designed materials. They have a self-executing ability to sense, evaluate and respond to external stimuli and can generate electricity by converting the kinetic energy [9]. From the perspective of global environment protection and sustainable development, exploring the evolution and application of deep-sea materials is important to alleviate the energy crisis and achieve carbon neutrality [10].

**Figure 1:**
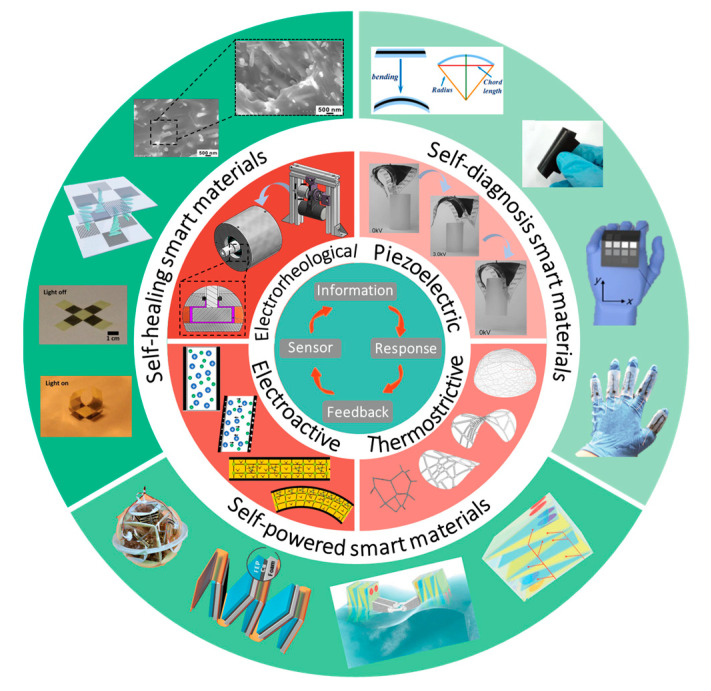
Scheme of the smart composite materials: mechanism, bionic design, and applications.

This article reviews the existing research results and limitations of marine composites and equipment or devices used for resources extraction. The rest of the review article is organized as: Section 2 summarizes the current research on the performance prediction, optimization design and fabrication processes of deep-sea composites, including both traditional and artificial intelligence methods, and the defects of existing methods are pointed out. By summarizing the advanced research results in the field of composite materials, the trend toward self-diagnosis and self-healing is proposed. Section 3 introduces two common types of deep-sea resources extraction, namely deep-sea underwater vehicle and deep-sea engineering (e.g., oil rig). The high demand for electrical energy supply of both the working process of deep-sea underwater vehicle and the monitoring equipment used for safety observation of engineering are pointed out, respectively. Thus, we put forward the future trend of developing deep-sea composites in the direction of self-powered energy. Based on the existing research and the demand for new composite materials in marine environment, we propose the concept of “deep-sea smart composite materials” in Section 4. We firstly present the functions of smart materials. Then, we introduce their concepts and roles of “self-diagnosis, self-healing, and self-powered”, respectively, and summarize existing research results. The scheme of smart composite materials is shown in Figure 1. Section 5 focuses on the research obstacles and trends of deep-sea smart composites. We present the challenges for self-diagnosis, self-healing, and self-powered materials, respectively, and point out the corresponding possible solutions. Then, based on the advanced properties and research challenges of these materials, it is proposed to progress more mature and advanced composites in conjunction with the development of artificial intelligence techniques, so as to apply them to individual components, integral equipment, and engineered structures in marine environment.

## 2. Deep-Sea Composite Materials

The primary demand for deep-sea structural materials is compressive resistance. There are three types of materials commonly used in deep-sea environment, namely, high-performance steels, alloys, and composites. Particularly, composites are the most widely used and promising materials due to their excellent compressive and corrosion resistance, water tightness, lightweight, and biological adhesion prevention [11]. High-performance steel has the advantages of high load-bearing capacity, easy processing, low cost, good fatigue strength, and energy absorption, but require precise control in their welding process. Alloy materials mainly include titanium, nickel, aluminum, and copper–nickel alloys, with low density, high strength, and corrosion resistance, but their surface corrosion-resistant passivation films are prone to corrosion cracking and environmental pollution under deep-sea high-pressure and low-density conditions [12,13]. The most commonly used composites are polymer and resin-based fiber-reinforced materials. The resin includes thermoplastic and thermosetting resin, and the reinforced fiber has carbon and glass fiber. Carbon fiber weights five times as much as glass fiber; however, carbon fiber owes high tensile strength and elastic modulus due to its lightweight. Other materials, such as high-strength ceramics, solid buoyancy materials, and protective coatings are commonly used for deep-sea exploitation as well [14,15].

### 2.1. A Brief Introduction of Composite Materials

Composite materials are a type of material that consists of a polymeric or metallic material, or a ceramic material as a matrix and a fiber or granular material as a reinforcement. Composite materials have many advantages for the use of deep-sea materials, such as lightweight, high strength, corrosion resistance and moisture resistance, and favorable fatigue performance [16]. The most commonly used matrices in marine environments are polymer and resin-based, while the reinforcements are fibers. The development of polymer-based fiber-reinforced composites is nearly maturity [17]. Due to the specific requirements for lightweight and corrosion resistance of materials in deep-sea, fiber-reinforced composites have been widely developed and applied for civil/military ships, offshore oil and gas extraction, and wind turbines [18]. The composition, structure and performance of composite materials are increasingly complicated due to the application requirements of different scenarios. The traditional models based on experimental observation, theoretical modeling and numerical simulation have encountered new scientific problems and technical bottlenecks in the design, analysis, and fabrication of deep-sea composite materials [19]. Insufficient experimental observations, lack of theoretical models, limited numerical analysis, and difficulties in result validation have restricted the future engineering applications of deep-sea composites to some extent [20]. The prediction, design, and fabrication of composites play a crucial role on their mechanical performance and application fields.

Advances in simulation models and practical applications are needed to move from the understanding of basic material properties to the development of quantitative methods that can interpret and predict experimental results [21]. In order to achieve accurate validation of composite simulations, computing systems should combine new theories and innovative applications that utilize powerful computational methods and infrastructures [22]. Advances in simulation and software will allow researchers to more realistically verify the complexity of composite materials [23].

### 2.2. Modern Intelligent Computing Methods in Composite Materials

Artificial intelligence (AI) techniques, such as deep learning (DL) and reinforcement learning (RL), have promising applications in composites as the analysis of big data and computational power increase [24]. Firstly, in terms of performance prediction, large numbers of data can be obtained through experimental tests and numerical simulations. By using these data, AI can extract complex relationships among high-dimensional variables and establish fast parametric and performance response [25]. Nosengo et al. [26] argued that candidate materials with typical desired properties can be quickly surfaced through computer modeling and machine learning. The amount of keyword search data on AI+ new materials are exploding. In addition, there is an urgent need to develop automated tools for processing and analyzing data. Secondly, there is no need to rely on experience or inspired intuition in optimal design. After setting an appropriate objective function, design strategies can be automatically updated for global optimization or exact inverse design [27]. Zhao et al. [28] proposed a material design system supported by AI and driven by data and introduced new material discovery and design method based on big data combined with AI and ML algorithms. This material system is designed for functional materials and covers semiconductor materials, dielectric materials, and metallic materials. Generative adversarial networks (GAN) have been successfully applied to inverse design, and deep reinforcement learning (DRL) has also emerged in optimal design [29,30]. Finally, in terms of fabrication processes, AI techniques will rapidly investigate the effects of various manufacturing parameters on the mechanical properties of composites and apply new techniques to large and complex structures through improved fabrication processes [31]. To a certain extent, these methods can reduce limitations of the dataset under a reasonable environment. It is expected that these methods can lead to the development of material optimization design models integrated with mechanical principles and the design of composite materials with various excellent properties [32]. Here, as shown in Table 1, the above three aspects of deep-sea composites are briefly reviewed in order to summarize the existent problems and report future trends of deep-sea composite materials and structures.

### 2.3. Performance Prediction

Traditional prediction methods for composite materials have many problems, such as the lack of theoretical models, incomplete numerical analysis, and difficulty in validating results, which seriously limit the rapid development of future applications of deep-sea composites [61]. Due to the complex and unique micro/nano-structural properties of deep-sea composites, three aspects of material, structure, and process should be considered in the design, which helps to achieve the best performance [62]. The prediction scheme of deep-sea composites, whether using FEM or other methods, can be summarized as follows. At first, the characteristic parameters of the material are needed to evaluate the material performance. Based on this, intelligent algorithms can be employed to update these parameters. Then, iterative processes are conducted until the properties cannot be improved [63].

**Figure 2:**
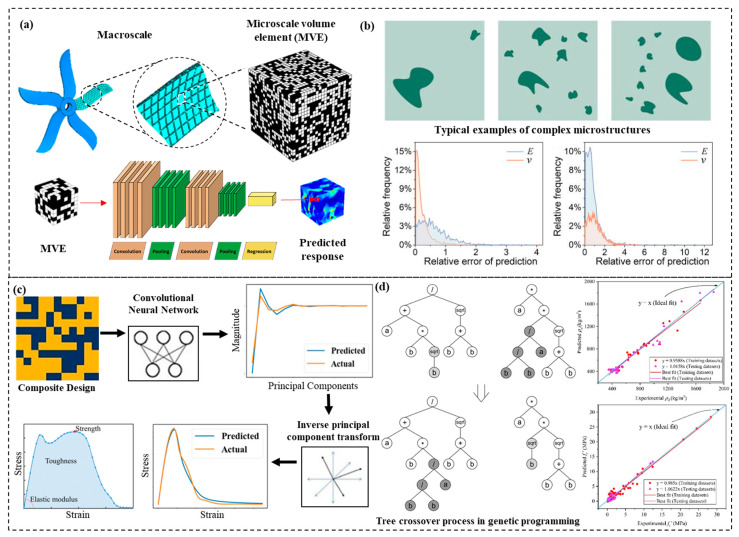
Current research on performance prediction of composite materials. (**a**) Localization linkages for elastic deformation of three-dimensional high contrast composites [67]. Copyright 2020 Elsevier. (**b**) DNN for predicting the mechanical properties of composites [65]. Copyright 2019 American Institute of Physics (AIP). (**c**) Prediction of composite microstructure stress–strain curves [66]. Copyright 2020 Elsevier. (**d**) An artificial intelligence-based gene expression programming approach was used for modelling the performance of bio-composites [68]. Copyright 2021 Elsevier.

AI-enabled methods are directly data-driven, eliminating the need for pre-built complex physical models or empirical parameters and transforming performance prediction from a traditional cause-and-effect relationships to an artificially intelligent variable mechanism [64]. The use of AI approaches to predict macroscopic mechanical properties based on microstructure images and design parameters can extract properties accurately and achieve good results in multi-scale mechanical prediction of composite materials [65,66], as shown in Figure 2b,c. In Figure 2a, Yang et al. [67] used 3D microstructures as the input, and the effective stiffness obtained from the finite element calculation is used as the output to train CNN model, and established the effective internal relationship between them. The CNN improved the prediction results by 54% accuracy compared with the conventional method. Ahmad et al. [68] proposed an AI-based gene expression programming approach to model the properties of bio-composites. A mathematical model of density, compressive strength, and thermal conductivity of bio-composites was proposed, as shown in Figure 2d, presenting a high degree of generalizability and predictability. Artificial intelligence algorithms still have to overcome the following issues in predicting the performance of deep-sea composites: (i) constructing sufficient experimental and numerical simulation databases, (ii) extracting key characteristic parameters to reduce the computational costs, (iii) quantifying the uncertainties and effects of parameters in the designed structure, (iv) introducing mechanical models and physical constraints in the AI-enabled prediction process, (v) implementing mechanical theory to the guidance of AI methods [69,70].

### 2.4. Optimization Design

The design of composite materials requires the preparation of materials with well-defined target properties based on summarized experimental laws and generalized scientific principles. Compared with an isotropic and homogeneous single materials, the mechanical properties and design requirements of composites are more complex, and empirical design methods are currently dominant [71,72]. Traditional design paradigm is trial-and-error, which usually relies on extensive experiments. This method requires lots of manpower, time, and resources through repeated experiments, while the final optimized design may not actually be the optimal one [73]. Hence, computer science is currently helping to design composite materials.

**Figure 3:**
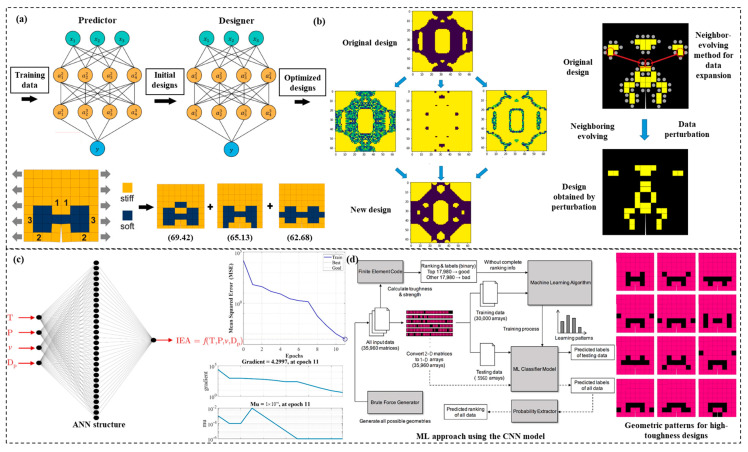
Current research on optimization design of composite materials. (**a**) Inverse materials design using backpropagation and active learning [76]. Copyright 2020 Elsevier. (**b**) Design of architectured composite materials with an efficient, adaptive artificial neural network-based generative design method [77]. Copyright 2022 Elsevier. (**c**) Development of an artificial neural network for predicting energy absorption capability of thermoplastic commingled composites [78]. Copyright 2021 Elsevier. (**d**) De novo composite design based on machine learning algorithm [79]. Copyright 2020 Elsevier.

The application of AI to design composite materials can effectively reduce the design cost and time [74]. Nowadays, with the maturity of numerical simulation technology and the continuous development of AI, it has become a reality to use computers for simulation experiments to explore more design options instead of research experiments, which provides an effective way and a new idea for accurate and efficient optimal design [75]. Herein, two antipodal approaches to material design can be used: a goal-oriented forward-optimized design and a demands-oriented reverse-optimized design, as shown in Figure 3a,b [76,77]. For example, as shown in Figure 3c, Qian et al. [78] developed an efficient artificial neural network-based inverse design method for designing architectural composites with novel properties. By employing adaptive learning and optimization strategies, the design space can be efficiently explored, thereby significantly reducing the amount of labeled training data required. As shown in Figure 3d, Chen and Gu [79] constructed an inverse design neural network to optimize the microstructure design of composites, containing two artificial neural networks. One can be trained with data to predict the performance; the other will use the weight matrix generated by the prediction network and output the optimal design solution instead of updating the weight matrix in the back-propagation stage.

Existing studies have shown that the use of AI-supported methods is an effective way to seek the optimal design path, depending on the influence of different micro/nano-scale parameters. However, the core of an intelligent computational approach lies in effective creation of material property datasets at an early stage [80,81]. Due to the complex multi-scale internal characteristics of deep-sea composites, a wide range of design parameters can be selected. Therefore, it remains a challenge to eliminate redundant features, find the most relevant optimal design parameters, and accurately quantify the uncertainties of design models to achieve an efficient optimization [82].

### 2.5. Fabrication Processes

**Figure 4:**
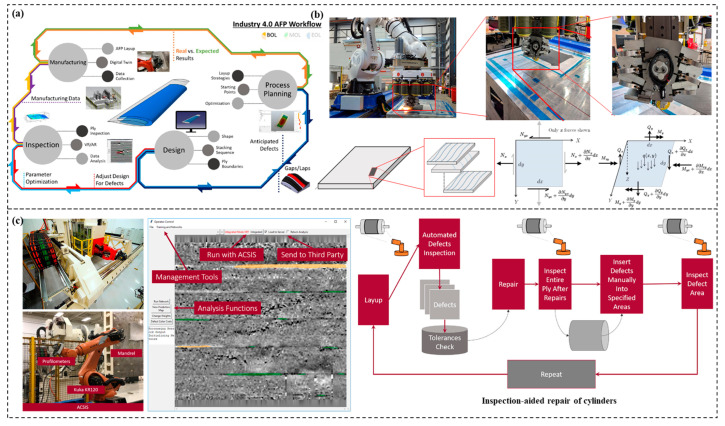
Current research on manufacturing application of composite materials. (**a**) Graphical representation of closed loop AFP process [85]. Copyright 2021 Elsevier. (**b**) Optimal fiber paths for robotically manufactured composite structural panels [87]. Copyright 2020 Elsevier. (**c**) AFP inspection in composites manufacturing [88]. Copyright 2020 Elsevier.

In practical applications, internal defects will significantly affect the mechanical properties of materials. During the manufacturing process, the use of sensors to detect their condition is beneficial to adjust relevant parameters and reduce damages of materials, so as to obtain expected mechanical properties [83]. During the molding process of deep-sea composites, appropriate parameters must be selected to evaluate the preferred material properties. Traditionally, the selection of molding parameters has been focused on the matrix; however, with industrialization, automated fiber placement (AFP), an advanced automated forming technique, is widely used to manufacture composites [84,85]. AFP can efficiently perform automated placement of complex curved surfaces with large curvature. Nevertheless, several factors can contribute to certain defects in the AFP process, such as lay-up speed and path, temperature, fiber tension, and so on. By introducing AI into AFP, defects in the forming processes can be found autonomously and fiber lay-up paths can be intelligently planned to further improve forming accuracy and efficiency [86]. For example, as shown in Figure 4a, Brasington et al. [85] proposed a novel approach for a closed-loop AFP circle, where the entire process has been composed of several isolated pillars: design, process planning, manufacturing, and inspection. Vijayachandran et al. [87] combined neural networks with genetic algorithm to optimize the flexural properties of composites (see Figure 4b). Sacco et al. [88] utilized full CNN and Marching Squares algorithm to drive robotic arms for repairing defects in composites, realizing the combination of AI and precision robotic system, as shown in Figure 4c.

Composite material processing has always been a problem. For example, precision machining, such as cutting and drilling, is prone to cause material damages, such as delamination, tearing and ablation, as well as severe tool wear. By introducing AI into deep-sea composites processing, thermal and mechanical problems, as well as processing parameters can be modeled and explored. In addition, real-time condition of tools and work pieces can be monitored and analyzed using AI-enabled methods to achieve dynamic sensing, judgement, and optimization [89]. Future developments will focus on studying the influence of various manufacturing parameters on composites properties, improving forming and processing techniques, and even cooperating with precision robotic systems to produce large complex composites structures [90].

### 2.6. Summary

In this section, we summarized the advanced research results on the performance prediction, optimization design and fabrication processes of deep-sea composites. The research methods for these three areas are divided into traditional methods and AI-supported methods. The trend of deep-sea composites materials toward self-diagnosis and self-healing is proposed based on deficiency of existing studies.

## 3. Composites in Deep-Sea Resources Exploration

There are usually two ways to explore deep-sea resources: one is to use deep-sea exploration equipment to obtain real-time data from designated areas of the ocean or seafloor by means of sensing or sampling. The second one is to construct large engineering structures, such as submarine space stations and offshore drilling platforms, to exploit deep-sea resources. Existing deep-sea exploration equipment includes three categories [91], namely, (i) deep-sea underwater vehicle (DUV) can carry varieties of electronic equipment, mechanical devices, and specialized personnel to reach all sorts of depth and environments quickly and accurately; (ii) sensing and detection technologies, including acoustic, optical, electromagnetic, and thermal sensing, are widely used in deep-sea data acquisition, navigation positioning, and target detection; (iii) sampling and detection techniques, such as biological sampling, seawater sampling, and core sampling [92,93]. Among them, the DUV technology is the main method of deep-sea exploration. However, due to the characteristics of poor visibility, high water pressure and complex topography of the marine environment, the development and application of DUV has always been the focus of scholars’ attention. Deep-sea exploration equipment needs long-term stable power supply during the working process. Similarly, engineering structures require real-time safety observation by using sensors and other monitoring devices in the course of their working process. These devices also need a long-term stable electricity in deep-sea environments [94,95]. Therefore, the study of abyssal self-powered materials is of great importance to solve these problems.

### 3.1. Two Applications of Composites in Exploration

Marine environments contain vast biological, energy, and metal resources, and DUV plays an irreplaceable role in the exploration of deep-sea resources. DUV includes three categories: human occupied vehicle (HOV), unmanned underwater vehicle (UUV), and other ocean survey equipment, such as deep-sea underwater gliders and towed mapping systems, etc. Among them, UUV, also known as underwater robot, is divided into remotely operated vehicle (ROV) and autonomous underwater vehicle (AUV) according to whether there is cable connection between the unmanned submersible and mother ship [96]. Cable controlled ROV can be divided into different types, such as floating, towed, crawling, and attached types, based upon its different movement modes. Non-cable controlled AUV can be categorized into pre-programming, monitoring type, and completely intelligent type from its intelligence level. With the development of UUV technology, some new UUVs have emerged in recent years, such as autonomous and remotely-operated vehicles (ARVs), which are a new type of UUV that combines some characteristics of ROV and AUV, with fiber optics for communication and power supply, and can be used as AUV without fiber optics, and has ROV function with fiber optics. In addition, a deep-sea exploration system also includes underwater glider, buoy automatic monitoring system, sonar, etc. [97].

Another method to exploit marine resources is to construct large deep-sea engineering. Structural health monitoring for these structures needs a large number of underwater navigation devices and buoys, such as underwater glider, deep-sea monitoring devices (DMDs), observation ROV, etc. It can provide real-time and accurate information for deep-sea resources exploration, management, and scientific research [98]. Currently, the power supply for DMDs mainly relies on batteries. In this review, the devices and equipment used for deep-sea SHM are collectively referred to as DMDs, which means no distinction is made here between sensing devices and detection devices [99,100]. Widely distributed DMDs face severe power supply challenges on account of the complex deep-sea environment and limited battery capacity. Due to the difficulties of fuel replenishment, exhaust gas emission and pressure bearing, DMDs put higher demands on power energy. Table 2 lists the state-of-the-art DUVs and DMDs with their materials composition, exploitation or sensing principles, and energy power supply.

### 3.2. Power Energy Sources for DUVs and DMDs

Electrical power supply in deep-sea environments should not only overcome the difficulties of high pressure, low temperature, and corrosion resistance, but also achieve the goals of high stability, controllability, and capacity and maintain low cost [117]. The current power energy sources for marine exploration mainly include batteries, such as lead-acid, silver-zinc, and nuclear energy, ocean thermal energy, as well as diesel fuel. Among them, the silver-zinc battery is commonly used as a power source with the advantages of high specific power and energy, safety, and stability. However, it also has the shortcomings of limited recharge times, short life span, and extremely high cost [118]. Lithium battery is the best comprehensive power energy source with the advantages of high voltage, strong capacity, long life, and fast charging. Large military submarines are usually powered by small nuclear energy units or closed-cycle diesel engines. Nuclear energy is advantageous on unlimited endurance, high safety, and long continuous working time [119]. However, the complexity of marine environments, limited battery capacity, and unsolved bio-attachment issues prevent large-scale deployment of DUVs and DMDs.

Blue energy is a clean, economical, and sustainable resource that can be converted from the ocean kinetic energy. There are two sources of blue energy, namely ocean currents and ocean waves. The kinetic energy is beneficial for powering distributed DMDs and small DUVs due to its availability and repeatability [120,121]. Considering the low power consumption of most DMDs, blue energy harvesting devices are expected to provide long-term efficient power supply. Currently, there are several advanced energy harvesting techniques to power marine exploration vehicles and monitoring devices [122,123]. Existing research and applications have demonstrated advances in small-scale energy harvesting and self-powered sensing devices, and can further improve ocean energy and ecological resource utilization to an unprecedented level.

Energy harvesting devices that are deployed on floating bodies or along cables can convert wave, solar, wind, or other renewable energy sources into usable electricity. For distributed sensing devices that require low power consumption and high repeatability, energy harvesting may be the most efficient approach for power supply [124]. Blue energy is expected to replace conventional batteries and seabed cables for future deep-sea power transmission. The converted energy can fully meet the needs of small and low-power electrical equipment and has the potential to be a stable long-term power source for deep-sea monitoring equipment. Blue energy resources are typically generated in areas of strong waves and currents; however, under realistic conditions, the interaction between waves, currents, winds, and temperature may reduce the efficiency of the associated energy conversion [125]. Given the instability and specificity of waves and currents, blue energy is still in its infancy and are expected to grow rapidly in the next decade.

### 3.3. Summary

This section introduces two common ways of deep-sea resources exploration, and focuses on the problem of supplying electrical energy to the equipment required for the extraction process. Traditional research approach lies in improving the energy density of the battery. Combined with the application of energy harvesting devices in ocean energy collection, blue energy is a feasible power supply solution. We illustrate the principles of harvesting blue energy from the ocean and claim that the problem of unstable wave and current energy harvesting still needs to be solved.

## 4. Deep-Sea Smart Composite Materials

In the process of deep-sea resources extraction by DUVs and marine engineering, cracks and fractures or other types of damage can occur due to the fatigue and aging of materials in harsh abyssal environments. With the expansion of cracks and fractures, the composite material splits could lead to the failure of composite structures. Traditional damage detection methods are limited by outdated equipment, low intelligence and poor timeliness, making it difficult to directly detect defects [126]. To avoid irreversible disasters caused by fatigue and aging, and to reduce manpower and financial costs required for periodic inspection, it is necessary to adopt deep-sea smart composites to meet the existing needs [127].

### 4.1. Main Function of Smart Materials

Two cores of smart materials are multifunctional composition and bionic design. Based on four mechanisms of sensing, feedback, response, and information recognition and accumulation, there are three main research directions of smart materials, including self-diagnosis, self-healing and self-powered [128]. For sensing, smart materials can perceive various changes in external and material self-conditions, such as load, stress, vibration, heat, light, etc. Feedback can be achieved by comparing the input and output information of the sensing system and providing the comparison results to the control system. Response can also be initiated by acting in a timely and dynamic manner based on the external and materials self-conditions. The sensing system performs identification by accumulating various information [129]. Based on prior mechanisms, self-diagnostic composites can be developed to solve problems, such as system failures and misjudgments, by analyzing and comparing system conditions with its past conditions [130]. Self-healing function is achieved by repairing damages through regenerative mechanisms, such as self-propagation, self-growth, and in situ reorganization. In terms of self-powered capability, on the one hand, the output of electrical signals can be used as active sensing; on the other hand, energy storage units and energy management modules can be integrated to obtain a sensing system that allows continuous real-time monitoring of state information without external power supply [131].

### 4.2. Self-Diagnosis Deep-Sea Composites

**Figure 5:**
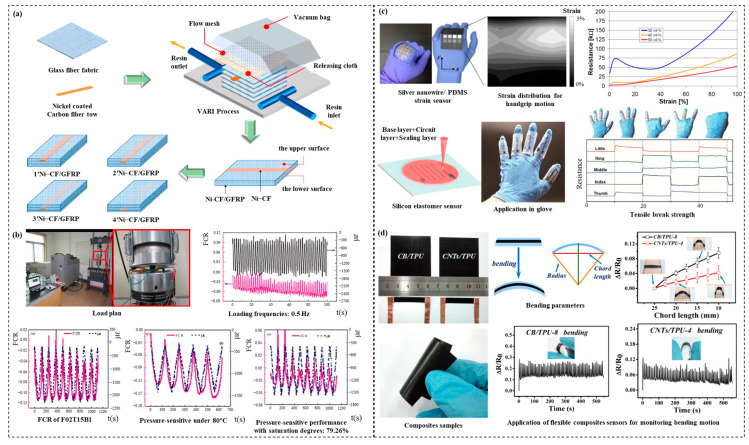
Existing applications of self-diagnosis, self-healing, and self-powered composite materials. (**a**) Polyurethane elastomers for crack self-diagnosis and healing tracking [135]. Copyright 2022 Elsevier. (**b**) Pressure sensitivity of multiscale carbon-admixtures–enhanced cement-based composites [136]. Copyright 2018 SAGE Publishing. (**c**) Elastomer strain sensor and its piezoresistive response for dynamic mechanical testing [138]. Copyright 2017 Elsevier. (**d**) Conductive thermoplastic polyurethane composites with tunable piezo-resistivity [137]. Copyright 2013 Elsevier.

Existing non-destructive testing (NDT) methods use sensing devices, such as X-ray, fiber optic, and acoustic emission sensors, but almost all conventional health monitors require knowledge of the damage area in advance, which is almost impossible for deep-sea environments [132,133]. However, self-diagnostic composites can sense resistance changes for overall real-time monitoring without the need for additional sensors. There are two methods to realize self-diagnostic composites. One is to implant sensors into the matrix of composites so as to collect damage signals and then assess the material or structural conditions. The other enables self-diagnosis without the need for additional sensors. For the former, the principle is to place conductive materials such as conductive fiber/nanoparticles, piezoelectric ceramic elements, and optical fibers in the matrix, thus forming a detection network that can conduct electricity. Then, the collected electrical or optical signals are analyzed to detect damage area and extent, thus enabling real-time monitoring of deep-sea composite materials and structures [134]. For the latter, large numbers of experiments have shown that damage detection can be effectively performed by incorporating conductive materials, such as carbon fiber-reinforced polymer (CFRP) in the matrix, which will contribute to the corresponding response on the resistance brought about by environmental changes, as shown in Figure 5a,b [135,136]. For glass fiber-reinforced polymer (GFRP) and other non-conductive materials, it is unlikely that changes in resistance can be detected directly; however, the addition of conductive nanofillers is a favorable way to build up the conductive network of the material by reducing the contact resistance between fibers, as shown in Figure 5c,d [137,138].

### 4.3. Self-Healing Deep-Sea Composites

**Figure 6:**
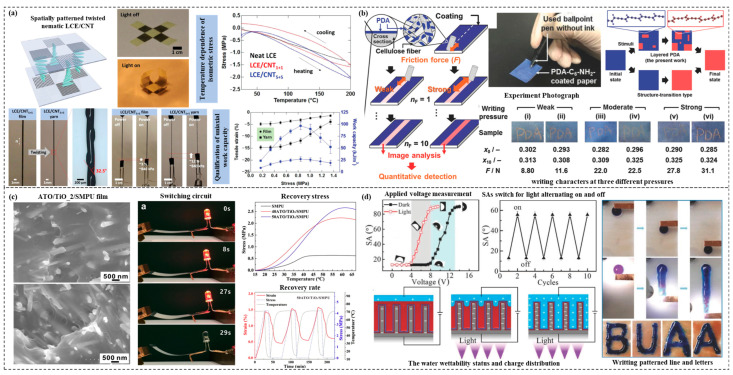
Existing applications of self-healing composite materials. (**a**) Actuating liquid crystal elastomer-carbon nanotube composites [143]. Copyright 2018 John Wiley and Sons. (**b**) Visualization and quantitative detection of friction force by self-organized organic layered composites [144]. Copyright 2019 John Wiley and Sons. (**c**) Electroactive shape memory composites for switching an electrical circuit [145]. Copyright 2021 John Wiley and Sons. (**d**) Photoelectric responsive nanoporous composites for droplets’ multifunctional manipulation [146]. Copyright 2018 John Wiley and Sons.

Self-healing composites have covered many fields, such as concrete, polymers, ceramics, metals, and so on. The damage patterns targeted by self-healing process include corrosion, fatigue, and other failure modes. Research on self-healing composites has mainly involved micro/macro structural design, fabrication system construction, structural performance assessment, and material mechanism prediction [139]. Self-healing materials can be classified into polymer-based, metal-based, and inorganic non-metallic-based types. The polymer-based type has intrinsic and extrinsic stimulation mechanisms. Metal-based bases focus mainly on engineered concrete and ceramics. In current research, polymer-based and metal-based fiber-reinforced composites have been used for deep-sea structures, with polymer-based being more established [140]. Due to the high brittleness and poor wear resistance of polymers, homogenous or heterogeneous cracking of material macromolecular chains can occur, generating microcracks that then lead to fractures and other failures [141]. Based on the mechanisms of action, polymer-based self-healing fiber-reinforced composites can be classified as intrinsic and extrinsic type. Intrinsic polymers use reversible reactions or chain segment movements of polymer molecules under external excitation to reorganize internal microstructure and achieve self-healing of micro-cracks. Such polymer-based self-healing fiber-reinforced composites can realize self-healing function for the damage situations, such as acid and alkaline environments, light, heat, and magnetic fields [142], but their applications are limited because the self-healing process cannot proceed spontaneously. Figure 6a,b illustrate that microcapsules and hollow fiber tubes of sealing repair agent are embedded in a polymer matrix, whose rupture can be triggered by micro-crack propagation, as a result, causing the release and curing of the repair agent, as well as the self-healing of damages [143,144]. Compared to the intrinsic type, the extrinsic type does not change the original chemical structure of polymers, and has better environmental resistance, wider range of use, and more diverse preparation systems and process schemes, as shown in Figure 6c,d [145,146]. However, they are rarely applied under practical conditions due to their difficulty in processing, long-term storage, and composition uniformity control. Meanwhile, micro-capsulated particles dispersed in a polymer matrix can reduce the mechanical properties of materials due to interfacial cleanliness and strength.

### 4.4. Self-Powered Deep-Sea Composites

**Figure 7:**
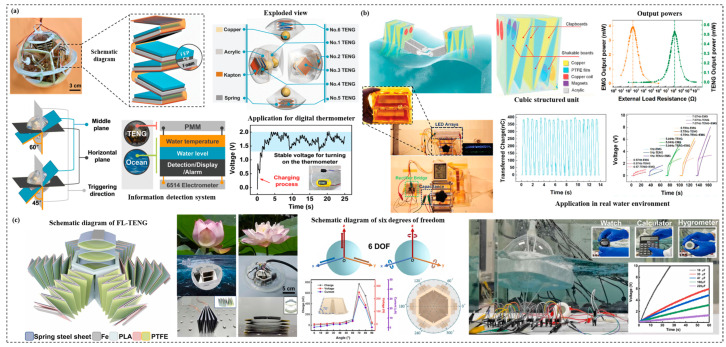
Present applications of self-powered composite materials. (**a**) Multidirectional water wave energy harvesting with power management [152]. Copyright 2020 Elsevier. (**b**) Optimized hybrid nanogenerator structure for wave energy harvesting [153]. Copyright 2019 John Wiley and Sons. (**c**) Flower-like triboelectric nanogenerator for blue energy harvesting with six degrees of freedom [154]. Copyright 2022 Elsevier.

Nanogenerators are new devices for converting and harvesting energy from natural environments. There are three types of nanogenerators, including piezoelectric nanogenerator, pyroelectric nanogenerator, and triboelectric nanogenerator (TENG) [147,148]. TENG is the most widely used and promising technology for applications of deep-sea composites, which utilizes the coupling effect of triboelectric and electrostatic induction between two materials with different electron gain and loss abilities. It can convert irregular low-frequency mechanical energy into usable electrical energy in human living environment. TENG has four basic working patterns, including vertical contact separation, horizontal sliding, single electrode, and independent friction layer [80,149]. Given its unique mechanism, TENG offers the advantages of superior output performance, unprecedented robustness, and universal applicability. Its applications cover biomedical and healthcare, chemical and environmental monitoring, smart transportation, smart cities, and energy harvesting from ocean waves [150,151]. At the same time, TENG offers an innovative way of harvesting large-scale blue energy from the ocean. For example, Figure 7a demonstrates that Liang et al. [152] proposed a spring-assisted multi-layer spherical blue energy harvesting device, based on the characteristics of TENG to collect low-frequency vibration energy, which can obtain wave energy from all directions. They also set a power management module to control the energy output. However, single energy harvesting technology cannot meet the demands of high-power deep-sea equipment. In order to achieve more efficient energy collection. As shown in Figure 7b, Wang et al. [153] developed a hybrid system, in which TENG complemented the functionality with an optimized internal topology. However, in the field of composites, current research focuses on ionic polymer-metal composites, which are characterized by lightweight, simple fabrication, low cost, good bending, and braking properties, as well as fast response, making them become an ideal choice for low-frequency energy acquisition in deep-sea. Figure 7c show that Wen et al. [154] designed and fabricated a flower-like TENG for kinetic energy harvesting with six degrees of freedom, which primarily collects kinetic energy with two degrees of freedom for horizontal motion and with three degrees of freedom for rotational motion.

### 4.5. Summary

The deep-sea smart composite materials proposed in this paper have three bionic functions “self-diagnosis, self-healing and self-powered”. The first two functions mainly aim at deep-sea composite materials, which can be used to construct deep-sea smart composite engineering and equipment to achieve the goal of self-diagnosis and self-healing. Self-powered property can be applied to provide continuous and stable electric energy for deep-sea exploration vehicles or monitoring devices. In summary, the deep-sea smart composite materials offer a new idea for deep-sea resources exploitation and scientific research.

## 5. Challenges and Prospects

### 5.1. Challenges

Since the self-diagnosis of marine composites is performed by installing conductive fibers or nanoparticles, piezoelectric ceramic elements, and optical fibers inside the matrix, a self-diagnostic network is formed. Then, real-time monitoring of composite components is realized by analyzing defect areas and damage degrees. Self-diagnostic composites can improve the safety and reliability of deep-sea exploration engineering and equipment, but there are disadvantages of complicated manufacturing and high cost, as well as the need to improve the detection sensitivity and environmental tolerance of composites. In addition, it is required to combine the damage mechanisms with self-diagnostic principles of composite materials to further optimize the detection schemes, accurately locating defect areas and determine damage degree.

After a considerable period of development, self-healing technologies still have some issues, such as high material costs, harsh external incentive conditions, and unclear in situ self-healing mechanisms. Hence, there is still a long way to go before large-scale engineering applications [155]. Future research on self-healing materials should mainly focus on the micro-structure design for typical failure patterns, the construction of new self-healing material systems, performance matching, and the evolutionary patterns of damage conditions, as well as dynamics and mechanisms of in situ damages self-healing [156]. Furthermore, we can implement active control and precise regulation of self-healing performance by further integrating real-time health condition sensing, rapid response and decision making, and in situ damages self-healing.

To solve the problem of powering deep-sea devices, conventional methods have been adopted to improve the energy density of batteries. Future research will concentrate on power enhancement and storage integration of energy harvesting devices [157]. By integrating energy harvesting and storage devices into a single system, self-charging power system (SCPS) can provide a continuous power supply. However, at present, SCPS is still in the proof-of-concept stage with hindrances, such as low integrated management efficiency and energy storage device selection, and research on different SCPS mechanisms is still ongoing. Nevertheless, the development of SCPS will help to solve problems of unstable wave and current energy harvesting and ensure long-term stable operation of DUVs and DMDs [158].

### 5.2. Prospects

We have summarized the existing research on deep-sea smart composites. The main advantages of the three bionic properties of marine smart materials are that they enable lightweight, miniaturized, mature, and stable abyssal exploration equipment. The self-diagnostic feature can monitor its own safety, and the self-healing characteristics can repair its own defects, which helps to realize stable operation of the equipment and structures for a long time in harsh environments. The self-powered feature does not need power supply units anymore, which will help to further reduce the size of the exploration equipment.

Deep-sea smart composites have a potential future in deep-sea exploration and innovative product development [159]. However, these composites are still in their early stage and have a long way ahead. In addition to overcoming the above-mentioned problems of bionic properties, the development of more advanced composite manufacturing processes will further reduce the cost of the equipment and engineering [160]. The most important issue is to achieve the use of marine bionic composites from labs to a wide range of practical applications.

In the near future, newer iterations of AI techniques, such as DL, and data mining by computer scientists will help to further investigate the development and applications of new smart composites. Experts in the field of composite materials will also combine these methods to explore more new smart composites in terms of performance prediction, optimization design, and fabrication processes. Additionally the development of marine smart composites enables the application from several components, such as propeller blades and ship hulls, to deep-sea exploration equipment and structural engineering. This has tremendous potential to extend the service life span of the equipment and reduce maintenance costs. Additionally, it is of great significance to help mankind better understand the deep-sea, so as to explore and develop resources in the extreme conditions.

## 6. Conclusions

Deep-sea composites play a significant role in deep-sea exploration technology. However, the development of existing deep-sea composites faces opportunities and challenges in three aspects, mainly in performance prediction, optimization design, and fabrication processes. Therefore, under the harsh deep-sea environment, it is of great importance to achieve self-diagnosis and self-healing of defects in the material itself. Corresponding to the specific application of the material can be divided into two categories, DUV and DMD. DUV is the main way of deep-sea exploration, DMD is used for real-time safety monitoring of deep-sea structures to ensure the safety and stability of the resource extraction process. In marine environment, both DUVs and DMDs require a long-term stable power supply.

To address these severe challenges of deep-sea exploration technology in terms of deep-sea composites, resources exploration vehicles, and monitoring devices, this article provides a perspective overview of deep-sea smart composite materials, a type of material with three bionic functions: self-diagnosis, self-healing, and self-powered. Deep-sea smart composites are important for risk assessment and prevention of DMDs and DUVs, as well as power supply, which has important implications for abyssal resources extraction. The self-diagnostic composites can sense resistance changes and allow for overall real-time monitoring without additional sensors. Self-healing composites can spontaneously repair in situ microcracks that cannot be detected in the early stages of the composite. Self-powered composites can collect energy from ocean currents and waves, and the collected blue energy can provide continuous and stable power for DUVs and DMDs. Finally, we summarize the research challenges of bionic functions and propose corresponding possible solutions. Combined with the development of AI techniques, we look forward to the development of new deep-sea smart composites and the popular application of these materials in marine equipment and structural engineering.

## Figures and Tables

**Table 1:** Summary for the recent research outcomes by means of composite materials, studied phenomena and corresponding methods.

Ref.	Application	Studied Phenomena	Method
[33]	Composites	Topology of base materials, toughness, strength	Finite element method (FEM), Linear, Convolutional neural networks (CNN)
[34]	Composites	Topology of base materials, stiffness	FEM, CNN
[35]	Hierarchical composites	Topology of unit cells, toughness, strength	FEM, CNN
[36]	Sphere, unsteady Stokes equations	Failure analysis, experimental testing parameters	Tensile test, Acoustic Emission, K-means (K-M)
[37]	Bi-directional woven fibers	Failure analysis, impact damage	Thermography, Artificial neural network (ANN), FEM simulations
[38]	Composite beams, plates, and shells	Design, optimization, and discovery	Machine learning (ML), Experimental, FEM
[39]	Laminated composite plates	Buckling resistance, stiffness, and strength	Genetic algorithm (GA), ANN, Simulated annealing (SA), Ant colony optimization (ACO)
[40]	Composite structures (functionally graded)	Stress distribution, critical buckling load, fundamental frequency	GA, Particle swarm optimization (PSO), ANN
[41]	Composites plates	Smart manufacturing	Deep learning (DL)
[42]	Composites	Materials design	ML
[43]	Composites tubes	Materials, processing, and structures engineering	DL, ML
[44]	Inorganic oxide, electrolyte, and metallic materials	Materials discovery and design	ML, Experimental
[45]	Composites	Design, optimization, properties prediction and discovery of materials	ML
[46]	Ti-2Al-9.2Mo-2Fe beta titanium alloy	Failure analysis, delamination	Ultrasonic, K-nearest neighbor (KNN), Decision tree (DT)
[47]	Lightweight foamed concrete	Experimental data, Damage mechanism analysis	Tensile test, Acoustic emission, Information coefficient (IC), KNN
[48]	Composite laminates	Experimental data, Design of fatigue-resistant composite	Hybrid algorithm, including GA and general phase-stepping algorithm (GPSA)
[49]	Glass fiber/matrix volume composites	Prediction of glass fiber/matrix volume fraction	Vibration-based test, ANN
[50]	Ferro cement composite structures	Damage mechanism analysis	Buckling test, Acoustic emission, K-M, Fuzzy c-means
[51]	Fiberglass-reinforced polyester composites	Experimental testing parameters, Prediction of fatigue lifecycles	Extreme learning machine (ELM), General regression neural network (GRNN)
[52]	Composite laminates	Experimental data, Buckling optimization	Hybrid algorithm, including GA, and general phase-stepping algorithm (GPSA)
[53]	Cylindrical shells	Buckling optimization	ANN, FEM simulations
[54]	Composite and sandwich plates	Construction of building trades directory, Experimental data	GA, ANN
[55]	3D woven composites	Multi-scale analysis and optimization	GA
[56]	Composite stiffened panels	Mechanical and hydrothermal loads	FEM simulations, GA, ANN
[57]	Polymer composites	Temperature control of microwave curing process	CNN, Experiment
[58]	Laminate stacking	Optimization	DT
[59]	Composite textiles	Optimization of manufacturing parameters and draping process	ANN, FEM simulations
[60]	Carbon Fiber-reinforced Plastics	Automated fiber placement processes	Levenberg–Marquarelt (LM), Experiment

**Table 2:** State-of-the-art DUVs and DMDs, and the materials, working principles, power supply categories.

DMD	Ref.	Major Material	Principles	Power Supply
AUV	[101]	Foaming material outside	Marine salvage and submarine construction operations	2000 W, rechargeable lithium-ion batteries
Pressure sensor	[102]	Composite materials	Measuring the pressure level and the water temperature.	0.7 W
Sonar	[103]	Steel, aluminum, titanium, composite, ceramic	Calculating echoes off the ocean bottom and floor	Average: 17 W; Max: 42 W, Silver-zinc, lead-acid, gel, alkaline lithium, nickel-cadmium batteries
Temperature sensor	[104]	Composite materials	Being mounted on Aanderaa Recording Instruments top-end plate	0.7 W; based on thermistor-bridge
ROV	[105]	Titanium alloy, glass fiber-reinforced composite	Aquaculture, underwater detection, physical and image sampling	>2000 W
Buoy	[106]	Polyethylene float. SS316 deployment tube	Plug-n-play, depending on sensor selections at time of purchase	27 W
Wave recorder	[107]	Composite materials	Being sampled and temperature compensated by an advanced Digital Signal Processor	0.7 W; based on a silicon piezoresistive pressure sensor
Conductive sensor	[108]	Composite materials	Being mounted in a String System node	1.4 W; the inductive principle
pH sensor	[109]	Composite materials	Ocean acidification, coral reef physiology and sensitivity, near-shore biological research	0.4 W (max); batteries, 5.4 kg (in air), 0.1 kg (in water)
Underwater glider	[110]	Titanium alloy, glass fiber-reinforced composite	Trailing a 10 cm long cylindrical antenna mounted on a 1 m stalk behind the main vehicle body	6.5 W; 81 D Lithium cells in 2 packs, Energy 10 MJ, Mass 9.4 kg
Chlorophyll	[111]	Xenoy, Lexan, Bronze, Titan, Edelstahl 316	Kor Interface Software, Bluetooth, Datenkabel, USB	0.24 W
Oxygen sensor	[112]	Composite materials	based on the ability of selected substances to act as dynamic fluorescence quenchers	1.4 W
Nitrate	[113]	Xenoy, Lexan, titanium, 316 stainless steel	KorEXO Software, RS-485, Mod Bus, USB, SDI-12	0.24 W
Glider payload	[114]	T-C Duct, pressure-protected thermistor	Conductivity, Temperature, Pressure, and up to seven auxiliary sensors	0.175 W; internal alkaline batteries (can be powered externally)
Turbidity sensor	[115]	Composite materials	RS-422, Simple Single Pair, 2400 Baud	0.12 W
Ultrasonic gauge	[116]	Sound velocities between 1000 and 9995 m/s	CygLink, Cygnus Topside Repeater	4.5 W, 550 g (19.4 oz)

## Data Availability

Not applicable.

## References

[1] Dover C. (2019). Inactive Sulfide Ecosystems in the Deep Sea: A Review. Front. Mar. Sci..

[2] Yan C., Ren X., Cheng Y., Song B., Li Y., Tian W. (2020). Geomechanical Issues in the Exploitation of Natural Gas Hydrate. Gondwana Res..

[3] Ruan X., Li X.-S., Xu C.-G. (2020). A Review of Numerical Research on Gas Production from Natural Gas Hydrates in China. J. Nat. Gas Sci. Eng..

[4] Shaibu R., Sambo C., Guo B., Dudun A. (2021). An Assessment of Methane Gas Production from Natural Gas Hydrates: Challenges, Technology and Market Outlook. Adv. Geo-Energy Res..

[5] Rosli N., Leduc D., Rowden A., Probert P. (2017). Review of Recent Trends in Ecological Studies of Deep-Sea Meiofauna, with Focus on Patterns and Processes at Small to Regional Spatial Scales. Mar. Biodivers..

[6] Huang H., Zhu C., Zhang W., Hao S., Zhang S., Leng J., Wei Y. (2020). Design and Analysis of the Multifunctional Oil-Injection Equipment for Deep-Sea Hydraulic Systems. IEEE Access.

[7] Jin C. (2021). Research Report on Technologies and Equipment for Exploitation of Marine Combustible Ice Resources. IOP Conf. Ser. Earth Environ. Sci..

[8] Chen S., Qiu L., Sun S., Yang J., Meng Q., Yang W. (2021). Research Progress on Corrosion of Equipment and Materials in Deep-Sea Environment. Adv. Civ. Eng..

[9] Jiao P.C. (2021). Emerging artificial intelligence in piezoelectric and triboelectric nanogenerators. Nano Energy.

[10] Hoang A., Pham V.V., Nguyen X.P., Nguyen H., Le Anh T. (2020). The Electric Propulsion System as a Green Solution for Management Strategy of CO_2_ Emission in Ocean Shipping: A Comprehensive Review. Int. Trans. Electr..

[11] Jiao P.C., Borchani W., Hasni H., Lajnef N. (2017). A new solution of measuring thermal response of prestressed concrete bridge girders for structural health monitoring. Meas. Sci. Tech..

[12] Morampudi P., Namala K.K., Gajjela Y., Barath M., Prudhvi G. (2021). Review on Glass Fiber Reinforced Polymer Composites. Mater. Today Proc..

[13] Barri K., Jiao P.C., Zhang Q.Y., Chen J., Wang Z.L., Alavi A.H. (2021). Multifunctional meta-tribomaterial nanogenerators for energy harvesting and active sensing. Nano Energy.

[14] Kundalwal S. (2017). Review on Micromechanics of Nano- and Micro-fiber Reinforced Composites. Polym. Compos..

[15] Hou J., Zou W., Li Z., Gong Y., Burnashev V., Ning D. (2020). Development and Experiments of an Electrothermal Driven Deep-Sea Buoyancy Control Module. Micromachines.

[16] Mortazavian V., Fatemi A. (2015). Fatigue Behavior and Modeling of Short Fiber Reinforced Polymer Composites: A Literature Review. Int. J. Fatigue.

[17] Bhaskar V., Kumar D., Singh K. (2017). Laser Processing of Glass Fiber Reinforced Composite Material: A Review. Aust. J. Mech. Eng..

[18] Roseman M., Martin R., Morgan G. (2016). Composites in Offshore Oil and Gas Applications. Mar. Appl. Adv. Fibre Reinf. Compos..

[19] Aboshio A., Uche O., Akagwu P., YE J. (2021). Reliability-Based Design Assessment of Offshore Inflatable Barrier Structures Made of Fibre-Reinforced Composites. Ocean Eng..

[20] Pham D.C., Narayanaswamy S., Qian X., Sobey A., Achintha M., Shenoi R. (2016). A Review on Design, Manufacture and Mechanics of Composite Risers. Ocean Eng..

[21] Sutherland L. (2018). A Review of Impact Testing on Marine Composite Materials: Part I—Marine Impacts on Marine Composites. Compos. Struct..

[22] Smith J., Nebgen B., Zubatyuk R., Lubbers N., Devereux C., Barros K., Tretiak S., Isayev O., Roitberg A. (2019). Approaching Coupled Cluster Accuracy with a General-Purpose Neural Network Potential through Transfer Learning. Nat. Commun..

[23] Bartok A., Kermode J., Bernstein N., Csányi G. (2018). Machine Learning a General-Purpose Interatomic Potential for Silicon. Phys. Rev. X.

[24] Warren J., Ward C. (2018). Evolution of a Materials Data Infrastructure. JOM.

[25] Ben Chaabene W., Flah M., Nehdi M. (2020). Machine Learning Prediction of Mechanical Properties of Concrete: Critical Review. Constr. Build. Mater..

[26] Nosengo N. (2016). Can Artificial Intelligence Create the next Wonder Material?. Nature.

[27] Dong R., Dan Y., Li X., Hu J. (2020). Inverse Design of Composite Metal Oxide Optical Materials Based on Deep Transfer Learning and Global Optimization. Comput. Mater. Sci..

[28] Zhao X.-G., Zhou K., Xing B., Zhao R., Luo S., Li T., Sun Y., Na G., Xie J., Yang X. (2021). JAMIP: An Artificial-Intelligence Aided Data-Driven Infrastructure for Computational Materials Informatics. Sci. Bull..

[29] Mao Y., He Q., Zhao X. (2020). Designing Complex Architectured Materials with Generative Adversarial Networks. Sci. Adv..

[30] Nassar M., Arunachalam R., Alzebdeh K. (2017). Machinability of Natural Fiber Reinforced Composites: A Review. Int. J. Adv. Manuf. Technol..

[31] Jiao P.C., Alavi A.H. (2021). Artificial intelligence-enabled smart mechanical metamaterials: Advent and future trends. Int. Mater. Rev..

[32] Liu Y., Zhao T., Ju W., Shi S. (2017). Materials Discovery and Design Using Machine Learning. J. Mater..

[33] Kerni L., Patnaik A., Kumar N. (2020). A Review on Natural Fiber Reinforced Composites. Mater. Today Proc..

[34] Yang Z., Yabansu Y., Al-Bahrani R., Liao W., Choudhary A., Kalidindi S., Agrawal A. (2018). Deep Learning Approaches for Mining Structure-Property Linkages in High Contrast Composites from Simulation Datasets. Comput. Mater. Sci..

[35] Gu G., Chen C.-T., Richmond D., Buehler M. (2018). Bioinspired Hierarchical Composite Design Using Machine Learning: Simulation, Additive Manufacturing, and Experiment. Mater. Horiz..

[36] Xu D., Liu P.F., Li J.G., Chen Z.P. (2018). Damage Mode Identification of Adhesive Composite Joints under Hygrothermal Environment Using Acoustic Emission and Machine Learning. Compos. Struct..

[37] Saeed N., Abdulrahman Y., Amer S., Omar M. (2019). Experimentally Validated Defect Depth Estimation Using Artificial Neural Network in Pulsed Thermography. Infrared Phys. Technol..

[38] Chen C.-T., Gu G. (2019). Machine Learning for Composite Materials. MRS Commun..

[39] Nikbakt S., Kamarian S., Shakeri M. (2018). A Review on Optimization of Composite Structures Part I: Laminated Composites. Compos. Struct..

[40] Nikbakht S., Kamarian S., Shakeri M. (2019). A Review on Optimization of Composite Structures Part II: Functionally Graded Materials. Compos. Struct..

[41] Wang J., Ma Y., Zhang L., Gao R., Wu D. (2018). Deep Learning for Smart Manufacturing: Methods and Applications. J. Manuf. Syst..

[42] Mosavi A., Rabczuk T., Varkonyi-Koczy A. (2018). Reviewing the Novel Machine Learning Tools for Materials Design. Adv. Intell. Syst. Comput..

[43] Dimiduk D., Holm E., Niezgoda S. (2018). Perspectives on the Impact of Machine Learning, Deep Learning, and Artificial Intelligence on Materials, Processes, and Structures Engineering. Integr. Mater. Manuf. Innov..

[44] Ma H., Wang J., Lu Y., Guo Y. (2019). Lightweight Design of Turnover Frame of Bridge Detection Vehicle Using Topology and Thickness Optimization. Struct. Multidiscipl. Optim..

[45] Antony P., Manujesh P., Jnanesh N. Data Mining and Machine Learning Approaches on Engineering Materials—A Review. Proceedings of the IEEE International Conference on Recent Trends in Electronics, Information, & Communication Technology (RTEICT).

[46] Li C.-L., Narayana P.L., Reddy N.S., Seong Woo C., Yeom J.-T., Hong J., Park C.H. (2019). Modeling Hot Deformation Behavior of Low-Cost Ti-2Al-9.2Mo-2Fe Beta Titanium Alloy Using a Deep Neural Network. J. Mater. Sci. Technol..

[47] Yaseen Z., Deo R., Hilal A., Abd A., Cornejo Bueno L., Salcedo-Sanz S., Nehdi M. (2018). Predicting Compressive Strength of Lightweight Foamed Concrete Using Extreme Learning Machine Model. Adv. Eng. Softw..

[48] Deveci H., Artem H. (2017). Optimum Design of Fatigue-Resistant Composite Laminates Using Hybrid Algorithm. Compos. Struct..

[49] Ezani F.I., Abdul Majid M.S., Paulraj M.P., Ahmadhilmi E., Fakhzan M., Gibson A.G. (2016). A Novel Vibration Based Non-Destructive Testing for Predicting Glass Fibre/Matrix Volume Fraction in Composites Using a Neural Network Model. Compos. Struct..

[50] Behnia A., Ranjbar N., Chai H.K., Masaeli M. (2016). Failure Prediction and Reliability Analysis of Ferrocement Composite Structures by Incorporating Machine Learning into Acoustic Emission Monitoring Technique. Constr. Build. Mater..

[51] Li J., Dawood R., Aldlemy M., Abdullah J., Yaseen Z. (2018). Fiberglass-Reinforced Polyester Composites Fatigue Prediction Using Novel Data-Intelligence Model. Arab. J. Sci. Eng..

[52] Deveci H., Aydin L., Artem H. (2016). Buckling Optimization of Composite Laminates Using a Hybrid Algorithm under Puck Failure Criterion Constraint. J. Reinf. Plast. Compos. Struct..

[53] Pitton S., Ricci S., Bisagni C. (2019). Buckling Optimization of Variable Stiffness Cylindrical Shells through Artificial Intelligence Techniques. Compos. Struct..

[54] Mantari J., Yarasca J., Canales F., Arciniega R. (2019). New Methodology for the Construction of Best Theory Diagrams Using Neural Networks and Multi-Objective Genetic Algorithm. Compos. B Eng..

[55] Fu X., Ricci S., Bisagni C. (2016). Multi-Scale Analysis and Optimisation of Three-Dimensional Woven Composite Structures Combining Response Surface Method and Genetic Algorithms. CEAS Aeronaut..

[56] Marín L., Trias D., Badallo P., Rus G., Mayugo J.A. (2012). Optimization of Composite Stiffened Panels under Mechanical and Hygrothermal Loads Using Neural Networks and Genetic Algorithms. Compos. Struct..

[57] Zhou J., Li Y., Li D., Wen Y. (2019). Online Learning Based Intelligent Temperature Control during Polymer Composites Microwave Curing Process. Chem. Eng. J..

[58] Wagner R., Köke H., Dähne S., Niemann S., Hühne C., Khakimova R. (2019). Decision Tree-Based Machine Learning to Optimize the Laminate Stacking of Composite Cylinders for Maximum Buckling Load and Minimum Imperfection Sensitivity. Compos. Struct..

[59] Pfrommer J., Zimmerling C., Liu J., Kärger L., Henning F., Beyerer J. (2018). Optimisation of Manufacturing Process Parameters Using Deep Neural Networks as Surrogate Models. Procedia CIRP.

[60] Brüning J., Denkena B., Dittrich M.-A., Hocke T. (2017). Machine Learning Approach for Optimization of Automated Fiber Placement Processes. Procedia CIRP.

[61] Daghigh V., Lacy T., Daghigh H., Gu G., Teimouri Baghaei K., Horstemeyer M., Pittman C. (2020). Machine Learning Predictions on Fracture Toughness of Multiscale Bio-Nano-Composites. J. Reinf. Plast. Compos. Struct..

[62] Wang K., Shriver D., Banu M., Hu S., Xiao G., Arinez J., Fan H.-T. (2017). Performance Prediction for Ultrasonic Spot Welds of Short Carbon-Fiber Reinforced Composites. J. Manuf. Sci. Eng..

[63] Zabihi O., Ahmadi M., Nikafshar S., Preyeswary K., Naebe M. (2017). A Technical Review on Epoxy-Clay Nanocomposites: Structure, Properties, and Their Applications in Fiber Reinforced Composites. Compos. B Eng..

[64] Miskin M., Jaeger H. (2013). Adapting Granular Materials through Artificial Evolution. Nat. Mater..

[65] Ye S., Li B., Li Q., Zhao H.-P. (2019). Deep Neural Network Method for Predicting the Mechanical Properties of Composites. Appl. Phys. Lett..

[66] Yang C., Kim Y., Ryu S., Gu G. (2020). Prediction of Composite Microstructure Stress-Strain Curves Using Convolutional Neural Networks. Mater. Des..

[67] Yang Z., Yabansu Y., Jha D., Liao W., Choudhary A., Kalidindi S., Agrawal A. (2018). Establishing Structure-Property Localization Linkages for Elastic Deformation of Three-Dimensional High Contrast Composites Using Deep Learning Approaches. Acta Mater..

[68] Ahmad M.R., Chen B., Dai J.-G., Kazmi S.M.S., Munir M.J. (2021). Evolutionary Artificial Intelligence Approach for Performance Prediction of Bio-Composites. Constr. Build. Mater..

[69] Elbaz Y., Furman D., Toroker M. (2020). Modeling Diffusion in Functional Materials: From Density Functional Theory to Artificial Intelligence. Adv. Funct. Mater..

[70] Chen L.-Q., Chen L.-D., Kalinin S., Klimeck G., Kumar S., Neugebauer J., Terasaki I. (2015). Design and Discovery of Materials Guided by Theory and Computation. NPJ Comput. Mater..

[71] Abueidda D., Almasri M., Ammourah R., Ravaioli U., Jasiuk I., Sobh N. (2019). Prediction and Optimization of Mechanical Properties of Composites Using Convolutional Neural Networks. Compos. Struct..

[72] Li J., Laghari R. (2019). A Review on Machining and Optimization of Particle-Reinforced Metal Matrix Composites. Int. J. Adv. Manuf. Technol..

[73] Mohan N., Senthil P., Vinodh S., Jayanth N. (2017). A Review on Composite Materials and Process Parameters Optimisation for the Fused Deposition Modelling Process. Virtual Phys. Prototyp..

[74] Singh A., Rosenkranz D., Ansari M.H.D., Singh R., Kanase A., Singh S., Johnston B., Tentschert J., Laux P., Luch A. (2020). Artificial Intelligence and Machine Learning Empower Advanced Biomedical Material Design to Toxicity Prediction. Adv. Intell. Syst..

[75] Dobrzanski L., Honysz R. (2010). Artificial Intelligence and Virtual Environment Application for Materials Design Methodology. Arch. Mater. Sci. Eng..

[76] Chen C.-T., Gu G. (2020). Generative Deep Neural Networks for Inverse Materials Design Using Backpropagation and Active Learning. Adv. Sci..

[77] Qian C., Tan R.K., Ye W. (2022). Design of Architectured Composite Materials with an Efficient, Adaptive Artificial Neural Network-Based Generative Design Method. Acta Materialia.

[78] Di Benedetto R.M., Botelho E.C., Janotti A., Ancelotti Junior A.C., Gomes G.F. (2021). Development of an Artificial Neural Network for Predicting Energy Absorption Capability of Thermoplastic Commingled Composites. Compos. Struct..

[79] Gu G.X., Chen C.T., Buehler M.J. (2018). De novo composite design based on machine learning algorithm. Extrem. Mech. Lett..

[80] Hanakata P.Z., Campbell D.K., Park H.S. (2014). An Ionic Polymer Metal Composite Based Electrochemical Conversion System in the Ocean. Int. J. Electrochem. Sci..

[81] Hanakata P., Cubuk E., Campbell D., Park H. (2020). Forward and Inverse Design of Kirigami via Supervised Autoencoder. Phys. Rev. Res..

[82] Guadagno L., Vietri U., Raimondo M., Vertuccio L., Barra G., De Vivo B., Lamberti P., Spinelli G., Tucci V., De Nicola F. (2015). Correlation between Electrical Conductivity and Manufacturing Processes of Nanofilled Carbon Fiber Reinforced Composites. Compos. B Eng..

[83] Heinecke F., Willberg C. (2019). Manufacturing-Induced Imperfections in Composite Parts Manufactured via Automated Fiber Placement. J. Compos. Sci..

[84] Smith R., Qureshi Z., Scaife R., El-Dessouky H. (2016). Limitations of Processing Carbon Fibre Reinforced Plastic/Polymer Material Using Automated Fibre Placement Technology. J. Reinf. Plast. Compos..

[85] Brasington A., Sacco C., Halbritter J., Wehbe R., Harik R. (2021). Automated Fiber Placement: A Review of History, Current Technologies, and Future Paths Forward. Compos. Part C Open Access.

[86] Wong M.M.I., Azmi A., Lee C., Mansor A. (2018). Experimental Study and Empirical Analyses of Abrasive Waterjet Machining for Hybrid Carbon/Glass Fiber-Reinforced Composites for Improved Surface Quality. Int. J. Adv. Manuf. Technol..

[87] Vijayachandran A., Davidson P., Waas A. (2020). Optimal Fiber Paths for Robotically Manufactured Composite Structural Panels. Int. J. Non-Linear Mech..

[88] Sacco C., Baz Radwan A., Anderson A., Harik R., Gregory E. (2020). Machine Learning in Composites Manufacturing: A Case Study of Automated Fiber Placement Inspection. Compos. Struct..

[89] Luo L., Zhang B., Zhang G., Li X., Fang X., Li W., Zhang Z. (2020). Rapid Prediction and Inverse Design of Distortion Behaviors of Composite Materials Using Artificial Neural Networks. Polym. Adv. Technol..

[90] Dan Y., Zhao Y., Li X., Li S.B., Hu M., Hu J.J. (2020). Generative Adversarial Networks (GAN) Based Efficient Sampling of Chemical Composition Space for Inverse Design of Inorganic Materials. NPJ Comput. Mater..

[91] Raj M., Thakre S., Annabattula R.K., Kanjarla A.K. (2021). Estimation of Local Strain Fields in Two-Phase Elastic Composite Materials Using UNet-Based Deep Learning. Integr. Mater. Manuf. Innov..

[92] Wu X., Gao Y., Wang Y., Fan R., Ali Z., Yu J., Yang K., Sun K., Li X., Yanhua L. (2021). Recent Developments on Epoxy-Based Syntactic Foams for Deep Sea Exploration. J. Mater. Sci..

[93] Wang H., Yu Y., Cai Y., Chen X., Chen L., Liu Q. (2019). A Comparative Study of State-of-the-Art Deep Learning Algorithms for Vehicle Detection. IEEE Intell. Transp. Syst. Mag..

[94] Vasilj J., Stancic I., Grujic T., Music J. (2017). Design, Development and Testing of the Modular Unmanned Surface Vehicle Platform for Marine Waste Detection. J. Multimed. Inf. Syst..

[95] Huvenne V., Bett B., Masson D.G., Le Bas T., Wheeler A. (2016). Effectiveness of a Deep-Sea Cold-Water Coral Marine Protected Area, Following Eight Years of Fisheries Closure. Biol. Conserv..

[96] Wu T., Tao C., Zhang J., Wang A., Zhang G., Zhou J., Deng X. (2019). A Hydrothermal Investigation System for the Qianlong-II Autonomous Underwater Vehicle. Acta Oceanol. Sin..

[97] Cong Y., Fan B.J., Hou D.D., Fan H.J., Liu K.Z., Luo J.B. (2019). Novel Event Analysis for Human-Machine Collaborative Underwater Exploration. Pattern Recognit..

[98] Pushpakumara B.H.J., Ginigaddara T. (2021). Development of a Structural Health Monitoring Tool for Underwater Concrete Structures. J. Constr. Eng. Manag..

[99] Wang F.R., Chen Z., Song G.B. (2021). Smart Crawfish: A Concept of Underwater Multi-Bolt Looseness Identification Using Entropy-Enhanced Active Sensing and Ensemble Learning. Mech. Syst. Signal Process..

[100] Jiao P.C., Egbe K.J., Xie Y.W., Nazar A.M., Alavi A.H. (2020). Piezoelectric Sensing Techniques in Structural Health Monitoring: A State-of-the-Art Review. Sensors.

[101] (2012). Reviews of Power Systems and Environmental Energy Conversion for Unmanned Underwater Vehicles. Renew. Sust. Energ. Rev..

[102] Pressure Sensor. https://www.aanderaa.com/productsdetail.php?Pressure-Sensor-11.

[103] Gothi A., Patel P., Pandya M. (2022). Underwater Robotics. ICT Intell. Appl..

[104] Temperature Sensor. https://www.aanderaa.com/productsdetail.php?Temperature-Sensor-12.

[105] Observation ROV System. http://www.robosea.org/rov.html.

[106] High-Quality Data Buoy. https://www.xylemanalytics.co.uk/db600-real-time-data-buoy/.

[107] Pressure Based Waves. https://www.aanderaa.com/productsdetail.php?Wave-and-Tide-Sensor-13.

[108] Conductivity Sensor. https://www.aanderaa.com/productsdetail.php?Conductivity-sensor-9.

[109] pHsensor. https://www.seabird.com/seafet-v2-ocean-pHsensor/product-downloads?id=54627921732.

[110] Rudnick D.L., Davis R., Eriksen C.C., Fratantoni D.M., Perry M.J. (2004). Underwater Gliders for Ocean Research. Mar. Technol. Soc. J..

[111] Chlorophyll Sensor. https://exocad.com/de/benutzerhandbuecher.

[112] Oxygen Sensor. Working Principle. https://www.aanderaa.com/productsdetail.php?Oxygen-Sensors-2.

[113] Nitrate. https://vdocument.in/exo-user-manual-ysi-librarydocumentsmanualsexo-user-manua-exo-user-manual.html.

[114] Glider Payload CTD. https://www.seabird.com/.

[115] Turbidity Sensor. https://pdf.directindustry.com/pdf/aanderaa-data-instruments-as/turbidity-sensor-4112/104571-382615.html.

[116] Ultrasonic Thickness Gauge. https://cygnus-instruments.com/.

[117] Li S., Liu J., Xu H.X., Zhao H.Y., Wang Y.Q. (2018). Research Status of Autonomous Underwater Vehicles in China. Sci. Sin. Inf..

[118] Kim Y.-S., Lee S.K., Chung H.-J., Kim J.-G. (2018). Influence of a Simulated Deep Sea Condition on the Cathodic Protection and Electric Field of an Underwater Vehicle. Ocean Eng..

[119] Meyer H., Roberts E., Rapp H., Davies A. (2019). Spatial Patterns of Arctic Sponge Ground Fauna and Demersal Fish Are Detectable in Autonomous Underwater Vehicle (AUV) Imagery. Deep Sea Res. Part I Oceanogr. Res. Pap..

[120] Liu G., Xiao L., Chen C., Liu W., Pu X., Wu Z., Hu C., Wang Z. (2020). Power Cables for Triboelectric Nanogenerator Networks for Large-Scale Blue Energy Harvesting. Nano Energy.

[121] Gao L., Shan L., Xie W., Chen X., Wu L., Wang T., Wang A., Yue C., Tong D., Lei W. (2020). A Self-Powered and Self-Functional Tracking System Based on Triboelectric-Electromagnetic Hybridized Blue Energy Harvesting Module. Nano Energy.

[122] Jiao P.C., Hasni H., Lajnef N., Alavi A.H. (2019). Mechanical Metamaterial Piezoelectric Nanogenerator (MM-PENG): Design Principle, Modeling and Performance. Mater. Des..

[123] Hasni H., Jiao P., Alavi A., Lajnef N., Masri S. (2017). Structural Health Monitoring of Steel Frames Using a Network of Self-Powered Strain and Acceleration Sensors: A Numerical Study. Autom. Constr..

[124] Benoist N., Morris K., Bett B., Durden J., Huvenne V., Le Bas T., Wynn R., Ware S., Ruhl H. (2019). Monitoring Mosaic Biotopes in a Marine Conservation Zone by Autonomous Underwater Vehicle. Conserv. Biol..

[125] Liu H., Wang Z., Shan R., He K., Zhao S. (2020). Research into the Integrated Navigation of a Deep-Sea Towed Vehicle with USBL/DVL and Pressure Gauge. Appl. Acous..

[126] Ansari T., Singh K., Azam M. (2018). Fatigue Damage Analysis of Fiber-Reinforced Polymer Composites-A Review. J. Reinf. Plast. Compos..

[127] Alavi A.H., Jiao P.C., Buttlar W., Lajnef N. (2018). Internet of Things-Enabled Smart Cities: State-of-the-Art and Future Trends. Meas..

[128] Trenfield S., Tan H., Awad A., Buanz A., Gaisford S., Basit A., Goyanes A. (2019). Track-and-Trace: Novel Anti-Counterfeit Measures for 3D Printed Personalised Drug Products Using Smart Material Inks. Int. J. Pharm..

[129] Christie M., Sun S., Deng L., Deng D., Ning D., Du H., Zhang S., Li W. (2018). A Variable Resonance Magnetorheological-Fluid-Based Pendulum Tuned Mass Damper for Seismic Vibration Suppression. Mech. Syst. Signal Process..

[130] Khoo C.K., Shin J.W. Designing with Biomaterials for Responsive Architecture: A Soft Responsive “Bio-Structural” Hydrogel Skin. Proceedings of the Education and research in Computer Aided Architectural Design in Europe Conference (36th).

[131] Jeffries K. (2013). Enhanced Robotic Automated Fiber Placement with Accurate Robot Technology and Modular Fiber Placement Head. SAE Int. J. Aerosp..

[132] Sridaran V.R., Boller C. (2020). NDT Approaches to Optimize Acoustics Based SHM Systems for Anisotropic Composite Structures. ACS Appl. Electron. Mater..

[133] Migot A., Ethaib S., Giurgiutiu V. Experimental Investigation of the Delamination Severity in a Composite Plate Using NDT and SHM Techniques. Proceedings of the Active and Passive Smart Structures and Integrated Systems XV.

[134] Bowlby L., Saha G., Afzal M.T. (2018). Flexural Strength Behavior in Pultruded GFRP Composites Reinforced with High Specific-Surface-Area Biochar Particles Synthesized via Microwave Pyrolysis. Compos. Part A Appl. Sci. Manuf..

[135] Zhao Y., Yan C., Xu H., Cai G., Jia H., Chen G., Imran A., Zhu Y. (2022). In-Situ Structural Health Self-Monitoring and Diagnosing of Glass Fiber Reinforced Plastics with Embedded Nickel Coated Carbon Fiber. Compos. Part B Eng..

[136] Hong Y., Li Z., Qiao G., Ou J., Cheng W. (2018). Pressure Sensitivity of Multiscale Carbon-Admixtures–Enhanced Cement-Based Composites. Nanomater. Nanotechnol..

[137] Georgopoulou A., Clemens F. (2020). Piezoresistive Elastomer-Based Composite Strain Sensors and Their Applications. ACS Appl. Electron. Mater..

[138] Zheng Y., Yilong L., Dai K., Mengran L., Zhou K., Zheng G., Liu C., Shen C. (2017). Conductive Thermoplastic Polyurethane Composites with Tunable Piezoresistivity by Modulating the Filler Dimensionality for Flexible Strain Sensors. Compos. Part A Appl. Sci. Manuf..

[139] D’Elia E., Barg S., Ni N., Rocha V., Saiz E. (2015). Self-Healing Graphene-Based Composites with Sensing Capabilities. Adv. Mater..

[140] Barrios S.U., Hernández M., Verdejo R., Lopez-Manchado M.A. (2020). Design of Rubber Composites with Autonomous Self-Healing Capability. ACS Omega.

[141] Oladele I., Omotosho T., Adediran A. (2020). Review Article Polymer-Based Composites: An Indispensable Material for Present and Future Applications. Int. J. Polym. Sci..

[142] Fan L., Rong M., Zhang M., Chen X. (2018). Repeated Intrinsic Self-Healing of Wider Cracks in Polymer via Dynamic Reversible Covalent Bonding Molecularly Combined with Two-Way Shape Memory Effect. ACS Appl. Mater. Interfaces.

[143] Kim H., Lee J.A., Ambulo C., Lee H., Kim S., Naik V., Haines C., Aliev A., Robles R., Baughman R. (2019). Intelligently Actuating Liquid Crystal Elastomer-Carbon Nanotube Composites. Adv. Func. Mater..

[144] Terada H., Imai H., Oaki Y. (2018). Visualization and Quantitative Detection of Friction Force by Self-Organized Organic Layered Composites. Adv. Mater..

[145] Xia Y., He Y., Zhang F., Liu Y., Leng J. (2021). A Review of Shape Memory Polymers and Composites: Mechanisms, Materials, and Applications. Adv. Mater..

[146] Keyu H., Heng L.P., Zhang Y.Q., Liu Y., Jiang L. (2018). Slippery Surface Based on Photoelectric Responsive Nanoporous Composites with Optimal Wettability Region for Droplets’ Multifunctional Manipulation. Adv. Sci..

[147] Wang Z., Jiang T., Liang X. (2017). Toward the Blue Energy Dream by Triboelectric Nanogenerator Networks. Nano Energy.

[148] Egbe K.J., Nazar A.M., Jiao P.C., Yang Y., Ye X.H., Wang H.P. (2021). Vibrational Turbine Piezoelectric Nanogenerators for Energy Harvesting in Multiphase Flow Fields. Energy Rep..

[149] Han Z., Jiao P., Zhu Z. (2021). Combination of Piezoelectric and Triboelectric Devices for Robotic Self-Powered Sensors. Micromachines.

[150] Matin Nazar A., Egbe K.-J., Jiao P., Wang Y., Yang Y. (2021). Magnetic Lifting Triboelectric Nanogenerators (Ml-TENG) for Energy Harvesting and Active Sensing. APL Mater..

[151] Zhang Q., Liang Q.J., Nandakumar D.K., Qu H., Shi Q.F., Alzakia F.I., Jie Tay D.J., Yang L., Zhang X.P., Suresh L. (2021). Shadow Enhanced Self-Charging Power System for Wave and Solar Energy Harvesting from the Ocean. Nat. Commun..

[152] Liang X., Jiang T., Liu G., Feng Y., Zhang C., Wang Z.L. (2020). Spherical Triboelectric Nanogenerator Integrated with Power Management Module for Harvesting Multidirectional Water Wave Energy. Energy Environ. Sci..

[153] Wang J.Y., Pan L., Guo H.Y., Zhang B.B., Zhang R.R., Wu Z.Y., Wu C.S., Yang L.J., Liao R.J., Wang Z.L. (2019). Rational Structure Optimized Hybrid Nanogenerator for Highly Efficient Water Wave Energy Harvesting. Adv. Energy Mater..

[154] Wen H., Yang P., Liu G., Xu S., Yao H., Li W., Qu H., Ding J., Li J., Wan L. (2021). Flower-like Triboelectric Nanogenerator for Blue Energy Harvesting with Six Degrees of Freedom. Nano Energy.

[155] Huynh T.-P., Sonar P., Haick H. (2017). Advanced Materials for Use in Soft Self-Healing Devices. Adv. Mater..

[156] Kim H.M. (2018). Electroactive Polymers for Ocean Kinetic Energy Harvesting: Literature Review and Research Needs. J. Ocean Eng. Mar. Energy.

[157] Zhang Q., Liang Q.J., Liao Q.L., Ma M.Y., Gao F.F., Zhao X., Song T.D., Song L.J., Xun X.C., Zhang Y. (2018). An Amphiphobic Hydraulic Triboelectric Nanogenerator for a Self-Cleaning and Self-Charging Power System. Adv. Funct. Mater..

[158] Johnson K., Jannasch H., Coletti L., Elrod V., Martz T., Takeshita Y., Carlson R., Connery J. (2016). Deep-Sea DuraFET: A Pressure Tolerant PH Sensor Designed for Global Sensor Networks. Anal. Chem..

[159] Rubino F., Nisticò A., Tucci F., Carlone P. (2020). Marine Application of Fiber Reinforced Composites: A Review. J. Mar. Sci. Eng..

[160] Roh D.H., Lee D., Lee Y.I., Park Y.B. (2021). Machine Learning Aided Design of Smart, Self-Sensing Fiber-Reinforced Plastics. Compos. Part C Open Access.

